# AUTHORS INDEX

**DOI:** 10.3400/avd.ai.21-01000

**Published:** 2021-12-25

## A

Abe, Noriyuki 64

Akai, Takafumi 39

Akita, Naohiro 388

Akutsu, Koichi 163

Alberti, Antonino 264﻿, 393

Alberti, Vittorio 264﻿, 393

Ando, Mizuki 415

Anh, Tuan Tran 231

Anju, Keisuke 376

Anzai, Hitoshi 277

Aoki, Michiko 68

Arahata, Masahisa 252

Araki, Yuichi 376

Arima, Takahiro 198

Ariya, Takumi 83

Asakura, Hidesaku 252

Ashikaga, Takashi 146

Azuma, Nobuyoshi 79﻿, 380

## B

Bakoyiannis, Chris N. 348

Bera, Sudipta 132

Bessho, Ryuzo 56

Bhunchet, Ekapot 376

Bisht, Navneeta 132

Borulu, Ferhat 185

Bottini, Oscar 315

Browne, Tom 19

## C

Calik, Eyupserhat 185

Canh, Pham Hong 267

Chandrasekar, Sadhana 5

Chen, Kerry L 256

Cheng, Ernest M 256

Chi, Yung-Wei 315

Chiba, Kiyoshi 75﻿, 139

Chomei, Syunya 400

Christoforou, Panagitsa D. 348

Chu, Francis 256

Cuong, Nguyen Ngoc 267

Cutrupi, Andrea 264

## D

Deguchi, Juno 334

Duc, Nguyen Minh 267

Dung, Pham-Thi Viet 267

## E

Enomoto, Sakae 404

Erkut, Bilgehan 185

## F

Franco, Gaetana 264

Fujii, Hiromichi 192

Fujii, Masahiro 56

Fujisue, Jun 362

Fujita, Motoki 273

Fujiyoshi, Toshiki 88

Fukui, Shinya 396

Funada, Ryuichi 236

Furuyama, Tadashi 11

## G

Georgopoulos, Sotirios 348

Gianesini, Sergio 315

Goyal, Vikas Deep 132

Guntani, Atsushi 122

Gupta, Vivek 132

## H

Hamaguchi, Mari 400

Hamamoto, Masaki 159﻿, 411

Hamano, Kimikazu 273

Hamuro, Mamoru 404

Handa, Kazuma 396

Hara, Nobuhiro 146

Harada, Ryo 52

Harada, Takasuke 273

Hasegawa, Yuto 83

Hashimoto, Munetaka 260

Hashimoto, Takuya 334

Hashino, Akira 249

Hayahi, Masanori 83

Hayashi, Hiroshi 163

Hayashi, Jun 384

Higa, Shotaro 168

Higa, Syotaro 415

Hirokawa, Masayuki 323

Hiromoto, Atushi 407

Hirooka, Kazunobu 376

Hiruma, Hiromitsu 75

Honda, Tasuku 362

Hong, Qiantai 5

Horii, Taiko 177

Horikawa, Takafumi 153

Hoshina, Katsuyuki 39﻿, 92﻿, 281﻿, 323

Howard, Adam 19

## I

Iba, Yutaka 60

Ichikawa, Akiko 236

Ichikawa, Shuichi 236

Ichikawa, Yuichi 46

Imaeda, Yusuke 198

Imasaka, Ken-ichi 249

Inaba, Yu 68

Inafuku, Hitoshi 415

Inaki, Yasuhiko 108

Inoue, Hideaki 46

Inoue, Ikuo 31

Inoue, Kentaro 11﻿, 112

Inoue, Taishi 400

Inoue, Takeshi 400

Inuzuka, Kazunori 23

Iraha, Tomotaka 139

Isaji, Toshihiko 39

Iseki, Yohei 56

Ishibashi, Hiroyuki 198

Ishida, Atsuhisa 181

Ishii, Yosuke 56﻿, 407

Ishikawa, Takeru 46

Isoda, Ryutaro 181

Isogai, Ako 198

Ito, Junji 168

Ito, Takaaki 323

Iwasaki, Toshiya 236

## J

Jalalzai, Izatullah 185

## K

Kakizawa, Yumi 396

Kamada, Keisuke 380

Kamada, Takeshi 52

Kamiya, Hiroyuki 380

Kanaoka, Yuji 181

Kaneda, Hideaki 31

Kaneko, Masakazu 146

Kasai, Mio 68

Kasuga, Yoshio 355

Katahashi, Kazuto 23

Katayama, Keijiro 159

Katayama, Yoshihiko 112

Kawaharada, Nobuyoshi 52

Kawakubo, Eisuke 122

Kawashima, Motoharu 362

Kayama, Takafumi 23

Kichikawa, Kimihiko 244

Kikuchi, Shinsuke 79﻿, 380

Kinoshita, Ryoji 376

Kise, Yuya 168﻿, 415

Kishimoto, Noriaki 192

Kitagawa, Atsushi 99

Kitahara, Mutsunori 396

Kitamoto, Shohei 177

Kiuchi, Ryuta 153

Kobata, Takashi 323

Kobayashi, Kimihiro 384

Kobayashi, Taira 159﻿, 411

Koizumi, Nobusato 88

Koizumi, Tomomi 31

Kokaguchi, Kyosuke 173

Komada, Takuya 112

Komai, Hiroyoshi 328

Komiyama, Hidenori 163

Komiyama, Nobuyuki 31

Komori, Kimihiro 92﻿, 281

Kondo, Nobuo 368

Kondo, Yasuo 71

Konidari, Marianna 348

Kordzadeh, Ali 19

Kotoku, Akiyuki 75

Kotsis, Thomas 348

Koya, Atsuhiro 79﻿, 380

Kuinose, Masahiko 181

Kumakura, Hisao 236

Kumamaru, Hiraku 92﻿, 281

Kumita, Shin-ichiro 56﻿, 163

Kuniyoshi, Yukio 415

Kurazumi, Hiroshi 273

Kurimoto, Yoshihiko 60

Kurisu, Kazuhiro 249

Kuroda, Yoshinori 384

Kuroda, Yosuke 52

Kurose, Shun 11

Kusagawa, Hitoshi 112

Kwan, Justin 5

## L

Lee, Tetsumin 146

Lemech, Lubomyr D 256

Linh, Le Tuan 267

Lo, Zhiwen Joseph 5

## M

Maeda, Tatuya 415

Malik, Muhammad Hammad 341

Malik, Muhammad Saad 341

Malik, Safdar Ali 341

Maruhashi, Takaaki 75﻿, 139

Maruyama, Ryushi 60

Maruyama, Yuki 198

Massara, Mafalda 264﻿, 393

Masuda, Noriyasu 372

Masuda, Takahiko 60

Matsubara, Susumu 108

Matsubayashi, Yuji 71

Matsumoto, Takuya 122

Matsunaga, Masashi 108

Matsuo, Yae 236

Matsuura, Sohei 39

Matsuyama, Katsuhiko 198

Memon, Muhammad Yousuf 341

Menegatti, Erica 315

Mii, Shinsuke 122

Mikami, Takuma 52

Mimura, Hidefumi 75

Mine, Takahiko 56

Minh, Thong Pham 231

Mitsui, Kentaro 146

Mitsuoka, Hiroki 198

Miwa, Senri 404

Miyachi, Hideki 163

Miyahara, Shunsuke 362

Miyairi, Takeshi 75﻿, 139

Miyake, Yoichiro 368

Miyama, Noriyuki 328

Miyamoto, Shinji 188

Miyashita, Kohei 368

Mizumoto, Masahiro 384

Mizuno, Hiroshi 46

Mizuno, Keisuke 388

Mizushima, Shohei 56

Mizutani, Satoshi 407

Mo, Makoto 1﻿, 323

Mori, Masaki 11

Morikage, Noriyasu 273

Morisaki, Akimasa 192

Morisaki, Koichi 11

Morishita, Eriko 252

Morita, Ichiro 181

Moriyama, Yoshihiro 376

Mukohara, Nobuhiko 362

Munakata, Hiroshi 64

Murakami, Akihiko 260

Murakami, Hirohisa 362

Muramatsu, Toshihiro 31

Murata, Tomohiro 407

Myouchin, Kaoru 244

## N

Nagano, Takaaki 168

Nagao, Toshihiko 99

Nagase, Masashi 146

Nagashima, Yoshinori 277

Nagata, Yasutoshi 146

Nakaema, Moriyasu 168﻿, 415

Nakagawa, Sayako 177

Nakai, Hidekazu 400

Nakai, Shingo 384

Nakajima, Katsuyuki 31

Nakajima, Yasuo 139

Nakashima, Kuniki 236

Nakayama, Ken 11

Naraoka, Syuichi 52

Naruse, Ena 23

Natsume, Kayoko 83

Nemoto, Masaru 270

Nemoto, Naohiko 277

Nguyen, Quyen Le 231

Nishi, Hiroyuki 396

Nishimaki, Hiroshi 75﻿, 139

Nishina, Dai 56

Nishioka, Naritomo 60

Nitta, Giich 146

Nomura, Yoshikatsu 362

Noto, Tatsunori 277

Nozato, Toshihiro 146

## O

Ochiai, Tomonori 384

Oga, Yuki 108

Ogawa, Tomohiro 323

Ogawa, Yukihisa 75﻿, 139

Ogino, Hitoshi 88

Ohba, Eiichi 384

Ohkura, Kazuhiro 83

Ohtake, Hiroshi 153

Oka, Eiichiro 163

Okada, Kenji 400

Okadome, Jun 122

Okata, Shinichiro 146

Okazaki, Takanobu 159

Okuda, Hiroko 79

Omura, Atsushi 400

Onozawa, Shiro 323

Oonuki, Masahiro 376

Orimoto, Yuki 198

Osako, Motohiko 68

Osawa, Takuya 388

Oshima, Marie 39

Oue, Kensuke 368

Ozawa, Masamichi 411

Ozu, Yasuhisa 112

## P

Power, Mark 256

Prionidis, Ioannis 19

Pua, Uei 5

Punamiya, Sundeep 5

## Q

Quang, Thai Duy 267

Quek, Lawrence Han Hwee 5

## R

Ramirez, Manfred J. 19

Rehman, Zia Ur 118

## S

Sadiq, Ilyas 341

Sakamoto, Kosuke 177

Sakamoto, Shun-Ichiro 407

Sakamoto, Tomohiko 396

Sakashita, Hideki 328

Sakata, Kimimasa 236

Sakon, Yoshito 192

Sakurai, Yuka 139

Samura, Makoto 273

Sanada, Junichiro 153

Sano, Masaki 23

Sato, Hiroko 260

Sato, Osamu 334

Sato, Rumiko 46

Satokawa, Hirono 323

Sayed, Tamer 19

Seguchi, Ryuta 153

Sharma, Shobhit 132

Sharma, Varsha 256

Shibata, Toshihiko 192

Shibuya, Shunsuke 260

Shimabukuro, Noriko 64

Shimizu, Azusa 46

Shimizu, Hideyuki 92﻿, 281

Shimizu, Takayuki 277

Shimizu, Takeshi 355

Shimizu, Tsuyoshi 355

Shimizu, Wataru 163

Shindo, Akira 108

Shintani, Tsunehiro 83

Shiose, Akira 249

Shirakawa, Yukitoshi 396

Shirasu, Takuro 39

Shirasugi, Nozomu 323

Shokoku, Shintaro 323

Shuto, Takashi 188

Sohgawa, Etsuji 192

Suehiro, Kotaro 273

Sugano, Norihide 323

Sugihara, Fumie 163

Sugiyama, Kayo 88﻿, 198

Sugiyama, Satoru 108﻿, 323

Suhara, Masamitsu 334

Suzuki, Kenji 407

Suzuki, Kojiro 198

Suzuki, Ryo 273

Suzuki, Tomoaki 71

## T

Tadokoro, Yu 270

Taguchi, Hidehiko 244

Tajima, Yuta 173

Takaesu, Satoru 277

Takahashi, Kazuki 380

Takahashi, Shinya 159

Takahashi, Yosuke 192

Takai, Kanako 328

Takasaki, Taiichi 159

Takashima, Noriyuki 71

Takayama, Katsutoshi 244

Takayama, Toshio 39

Takayama, Yutaka 270

Takei, Hidehiro 79

Takeuchi, Hiroya 23

Tamate, Yoshihisa 260

Tamura, Shozo 99

Tan, Glenn Wei Leong 5

Tanaka, Hideyuki 188

Tanaka, Hiroshi 362

Tanaka, Hiroyuki 376

Tanaka, Rica 46

Tanaka, Satofumi 368

Tanaka, Toshihiro 244

Tarui, Tatsuya 153

Thi, Quynh Mai 231

Tochikubo, Ai 380

Todoroki, Hirona 388

Tonoki, Shuto 362

Tsujimoto, Takanori 400

Tsukagoshi, Junji 60

Tsuruta, Ryosuke 273

## U

Uchida, Daiki 380

Uchida, Kaoru 188

Uchida, Tetsuro 384

Uchino, Gaku 362

Uchiyama, Hidetoshi 376

Uchiyama, Hiroki 52

Uejo, Akino 168

Ueno, Yasutaka 249

Unno, Naoki 23

Usta, Hakan 185

Uwabe, Kazuhiko 372

## V

Volpe, Pietro 264﻿, 393

## W

Wada, Takeshi 244

Wada, Tomoyuki 188

Wakisaka, Hodaka 71

Watanabe, Go 153

Watanabe, Tatsuki 270

Watanabe, Tatsuya 181

Watanabe, Yukihiro 163

## Y

Yaguchi, Tomoyuki 277

Yamabe, Kentaro 68

Yamada, Akira 60

Yamada, Norikazu 1

Yamada, Shinya 252

Yamaki, Takashi 323

Yamamoto, Junji 270

Yamamoto, Kenji 404

Yamamoto, Mai 99

Yamamoto, Naoto 23

Yamamoto, Nobuko 328

Yamamoto, Satoshi 334

Yamamoto, Yohei 376

Yamanaka, Katsuhiro 400

Yamanaka, Yuta 23

Yamane, Kokoro 192

Yamashiro, Satoshi 168

Yamashita, Atsushi 384

Yamashita, Sho 11

Yamashita, Yoichi 177

Yamashita, Yugo 1

Yamazato, Takahiro 64

Yanagawa, Naoki 260

Yanase, Yosuke 60

Yasugi, Takumi 323

Yasui, Daisuke 163﻿, 407

Yasumori, Ken 64

Yata, Tatsuro 23

Yee, Juliana 256

Yeo, Clarice Biru 5

Yong, Enming 5

Yoshiga, Ryosuke 122

Yoshimura, Yusuke 407

Yoshino, Shinichiro 11

Yuhn, Changyoung 39

## Z

Zulifqar, Muhammad Bin 341

